# SARS-CoV-2 Antibody Dynamics after COVID-19 Vaccination and Infection: A Real-World Cross-Sectional Analysis

**DOI:** 10.3390/vaccines11071184

**Published:** 2023-06-30

**Authors:** Ritthideach Yorsaeng, Kamolthip Atsawawaranunt, Nungruthai Suntronwong, Sitthichai Kanokudom, Jira Chansaenroj, Suvichada Assawakosri, Pornjarim Nilyanimit, Ratchadawan Aeemjinda, Nongkanok Khanarat, Lakkhana Wongsrisang, Chompoonut Auphimai, Preeyaporn Vichaiwattana, Sirapa Klinfueng, Thanunrat Thongmee, Donchida Srimuan, Thaksaporn Thatsanathorn, Natthinee Sudhinaraset, Nasamon Wanlapakorn, Yong Poovorawan

**Affiliations:** 1 Center of Excellence in Clinical Virology, Department of Pediatrics, Faculty of Medicine, Chulalongkorn University, Pathum Wan, Bangkok 10330, Thailand; ritthideach.yor@gmail.com (R.Y.); dr_natthinee@hotmail.com (N.S.);; 2 Institute for Urban Disease Control and Prevention, Department of Disease Control, Ministry of Public Health, Anusawari, Bang Khen, Bangkok 10220, Thailand; 3 The Royal Society of Thailand, Sanam Sueapa, Dusit, Bangkok 10300, Thailand

**Keywords:** SARS-CoV-2, COVID-19, vaccine, booster, anti-RBD, humoral immunity, infection

## Abstract

The Coronavirus disease 2019 (COVID-19) pandemic, caused by Severe Acute Respiratory Syndrome Coronavirus 2 (SARS-CoV-2), continues to surge despite the widespread use of vaccination. In Thailand, more than 77% and 39% of the population received two doses and three doses of COVID-19 vaccines as of December 2022, respectively. In addition, during the Omicron predominant period in 2022, more than 70% of Thai individuals have been infected. To gain comprehensive insight into SARS-CoV-2 antibody dynamics following vaccination or following vaccination and infection (hybrid immunity), we performed a cross-sectional analysis of sera samples from individuals who received COVID-19 vaccination and/or have been infected with COVID-19 in Thailand between January 2021 and December 2022. A total of 4126 samples were collected. Humoral immunity was evaluated by quantifying the immunoglobulin (including IgG, IgM, and IgA isotypes) specific to the SARS-CoV-2 receptor-binding domain (RBD) or Ig anti-RBD. The results showed that individuals who received two-dose vaccination alone had lower levels of Ig anti-RBD, which rapidly waned over time. To restore the waning antibody, a third dose vaccination is recommended for uninfected individuals who have only received 2 doses.

## 1. Introduction

Since the emergence of Severe Acute Respiratory Syndrome Coronavirus 2 (SARS-CoV-2) in 2019 [1], the virus has continued to evolve, resulting in new waves of infection. As of March 2023, COVID-19 has infected over 731 million individuals worldwide [2], resulting in 6.7 million deaths. In Thailand, there were reports of over 4.7 million cases and 33.7 thousand deaths due to COVID-19 as of March 2023 [3].

Vaccines can mitigate the disease severity but may not always prevent infection [4,5]. Several vaccine platforms have been developed and implemented worldwide to combat the disease. Currently approved vaccines include inactivated viruses, viral vector mRNA, and protein subunit vaccines. Several studies reported high incidence of breakthrough infections due to the immune escaping Omicron variants [6,7] and waning immunity over time. There were reports suggesting that immunity induced by two-dose vaccination waned over time, and third-dose booster vaccination is required to increase protection [8,9]. Besides, hybrid immunity induced by vaccination and infection has been shown to have durable protection and induce a high level of humoral immune response [10].

In Thailand, the COVID-19 vaccination campaign was initiated on 28 February 2021. Due to the limited vaccine availability and low transmission at that time, vaccination was initially prioritised for nationwide at-risk medical personnel, patients with underlying diseases (chronic respiratory disease, cardiovascular disease, chronic kidney disease, cerebrovascular disease, obesity, cancer and diabetes mellitus), and people who lived in the high-risk areas (Western Bangkok and Samut Sakhon). Vaccines that were mainly used in Thailand during the early phase were CoronaVac (Sinovac Biotech, Beijing, China) and ChAdOx1-S (AstraZeneca-University of Oxford, Oxford, UK), which were prioritized for those ≥60 years old. In July 2021, Thailand encountered a scant vaccine supply. The “mix and match” (heterologous prime) regimen was implemented in July 2021. The benefit of the heterologous prime-boost regimen (CoronaVac-ChAdOx1-S) is shortening the interval between ChAdOx1-S from 10 weeks to 4 weeks with similar antibody levels [11,12]. Moreover, Thailand implemented a booster vaccination in July 2021 to increase immunity in two-dose inactivated vaccine recipients. Booster vaccination used at that time was primarily ChAdOx1-S which could induce humoral immunity against the Delta variant circulating at that time [13,14,15]. The mRNA platform, BNT162b2 (Pfizer-BioNTech, New York, NY, USA), was introduced to Thailand in August 2021, and mRNA-1273 (Moderna-NIAID, Cambridge, MA, USA) later in November 2021. The mRNA vaccines were used mainly for booster shots to generate immunity for emerging variants [16,17,18,19].

The antibody levels correlate positively with vaccine efficacy [20]. Although the COVID-19 vaccines are effective against symptomatic and severe COVID-19 disease [5], immunity wanes over time. The effectiveness of two-dose vaccination remains highly protective against severe diseases for about 5–6 months [21]. The waning immunity could reduce the protection of the host. After the third dose of vaccination, immunity substantially increased [13,14,15]. Recent studies have shown that the fourth dose of vaccine can induce a booster response in the three-dose vaccinated individuals regardless of which type of vaccine was given for the first three doses [22].

As of February 2023, 85% of the Thai population had received at least two doses of the COVID-19 vaccine, while 80% had received three doses, and 40% had received four doses. According to a study in Chonburi province in December 2022, approximately 72% of the study population has been infected with COVID-19 as defined by seropositivity of antibody against nucleocapsid protein [23]. Therefore, it is likely that the majority of Thai population now has hybrid immunity. In a large study conducted in Israel, protection induced by previous infection was higher than that conferred by the two-doses vaccination in previously uninfected persons [24]. Besides, the rapid decline of SARS-CoV-2 antibody were more pronounced among the vaccinated non-infected individuals than those previously infected [25,26].

To provide antibody level dynamics and determine the eligible candidates for booster vaccination among people residing in Thailand, we conduct a cross-sectional study in a real-world situation in individuals receiving varying doses (2, 3, 4 doses) of COVID-19 vaccine with or without natural infection. The finding will assist in providing guidelines and planning for booster vaccine administration.

## 2. Materials and Methods

### 2.1. Study Design

This study was a cross-sectional study that included participants who sought SARS-CoV-2 antibody testing by serological assay at the research unit of the Center of Excellence in Clinical Virology, Faculty of Medicine, Chulalongkorn University, Bangkok, Thailand, between January 2021 and December 2022. A research nurse administered an on-site questionnaire to obtain information including age, sex, underlying diseases, blood collection date, COVID-19 vaccine type, vaccination date, and history of infection by RT-PCR or antigen test. Inclusion criteria for data analysis included individuals who received two to four doses of COVID-19 vaccination with known vaccine type and the date of vaccination, and those who had a sampling interval between the last dose of vaccine and blood sampling of ≥14 days. Participants with continuing or history of cancer, immunosuppressive therapy, and immunocompromised host were excluded. This study protocol was approved by the Institutional Review Board of the Faculty of Medicine of Chulalongkorn University (IRB numbers 546/64 and 99/65). As all the data collected for final analysis in this study has been anonymized, the institutional review board of the Faculty of Medicine, Chulalongkorn University has waived the need for written informed consent.

### 2.2. Serologic Assessment

A blood sample was collected by venepuncture into the clotted activator blood collection tube. All specimens were tested for SARS-CoV-2 antibody within the same day or kept at 4 °C for no more than 3 days using Elecsys SARS-CoV-2 S (Cat. no. 09289267190, Roche Diagnostics, Basel, Switzerland) performed on the Cobas e 411 analyzer (Cat. no. 04775201001, Roche Diagnostics, Basel, Switzerland) to detect the immunoglobulin (including IgG, IgM and IgA isotypes) against the SARS-CoV-2 targeting receptor-binding domain (RBD) or Ig anti-RBD. This system is based on Electrochemiluminescence immunoassay (ECLIA). The results of ≥0.8 U/mL were considered seropositive according to the manufacturer’s instruction.

### 2.3. Statistical Analysis

Statistical analysis used GraphPad Prism version 9.3.1 (GraphPad, San Diego, CA, USA) and IBM SPSS Statistics version 26 (IBM, Armonk, NY, USA). The category values were reported as numbers with percentages. The continuous values were reported as appropriate central values with dispersions. Fisher’s Exact calculated the difference between group characteristics in category values. The continuous values (antibody level) were log-transformed. The interval to sampling was stratified by month ±0.5; 14–45 days (1 month), 46–75 days (2 months), 76–105 days (3 months), 106–135 days (4 months), 136–165 days (5 months), and >165 days (>5 months). Comparison between groups was calculated by one-way ANOVA or Kruskal-Wallis. A Bonferroni adjustment or Dunn’s was used for multiple comparisons. The tests were used depending on the data distribution. A log-transformed data was used and converted to a geometric mean ratio (GMR) with a Bonferroni adjustment. We used a one-phase exponential decay assumption to create the trend lines for the relationship between the antibody and the interval after the last dose. In uninfected participants, the interval after the last dose was extended to 165 days for line smoothness and reliability.

## 3. Results

### 3.1. Demographic Data of Study Participants

**Figure 1:**
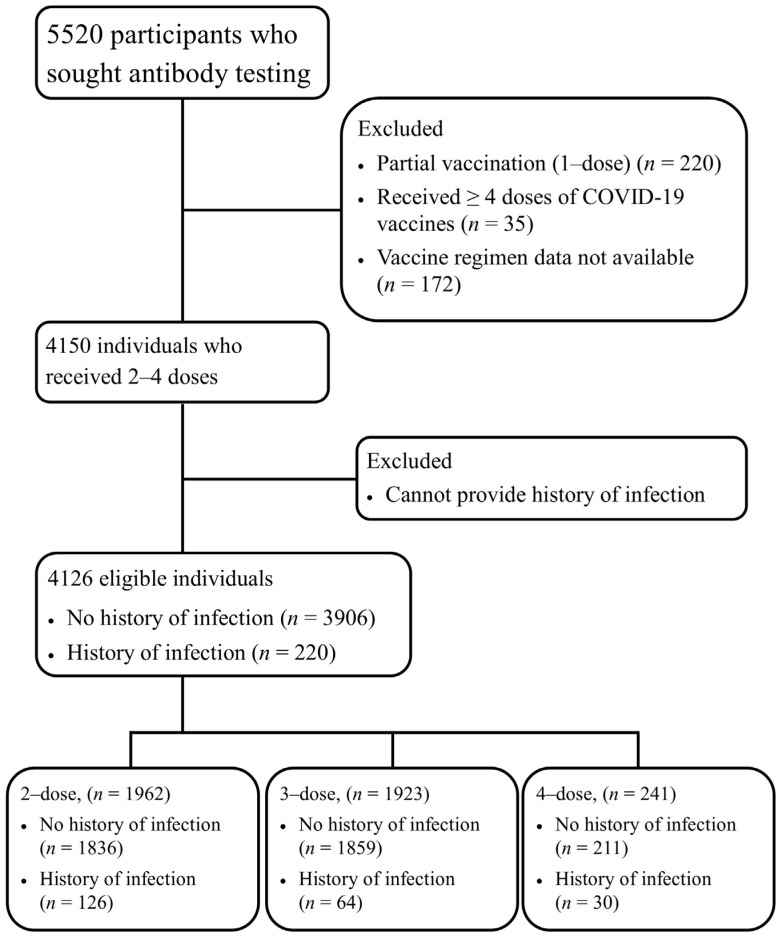
Flow chart of this study.

A total of 4126 individuals were enrolled in the study. The consort flow diagram is shown in Figure 1. Individual demographic data were classified by dose, i.e., 2-dose, 3-dose, and 4-dose vaccination, as shown in Table 1. A total of 3906 had no history of infection, and 220 had a history of infection based on RT-PCR or antigen test. Various COVID-19 vaccine platforms were used among participants, including inactivated (BBIBP-CorV, Sinopharm, Beijing, China, and CoronaVac, Sinovac Biotech, Beijing, China), adenoviral vector (ChAdOx1-S, AstraZeneca-University of Oxford, Oxford, UK), protein subunit (NVX-CoV2373, Novavax, Gaithersburg, MD, USA), and mRNA (BNT162b2, Pfizer-BioNTech, New York, NY, and mRNA-1273, Moderna-NIAID, Cambridge, MA, USA). There were 1962 individuals who received 2-dose vaccination, 1923 who received 3-dose, and 241 who received 4-dose vaccination.

The interval between last dose vaccination and blood sampling (time since last dose vaccination) ranged between 14 days to 376 days (median: 33 days, IQR; 28, 56). A small group (*n* = 122) of non-infected individuals with time since last dose of over 165 days were excluded for the trendline smoothness as described in the method. Nevertheless, data on the interval between the previous infection and blood sampling were not available.

### 3.2. Immunity in Participants with No Previous Infection

There was a total of 3906 participants who had no history of infection. Among the 3906 vaccinated uninfected participants, the median (IQR) Ig anti-RBD level was 173.4 (160.9, 186.9) U/mL after the second dose, rising to 7599 (7249, 7967) U/mL after the third dose and 8199 (7025, 9568) U/mL after the fourth dose (Table 1). The geometric mean titers of Ig anti-RBD after the third and fourth dose was significantly higher than after the second dose.

**Figure 2:**
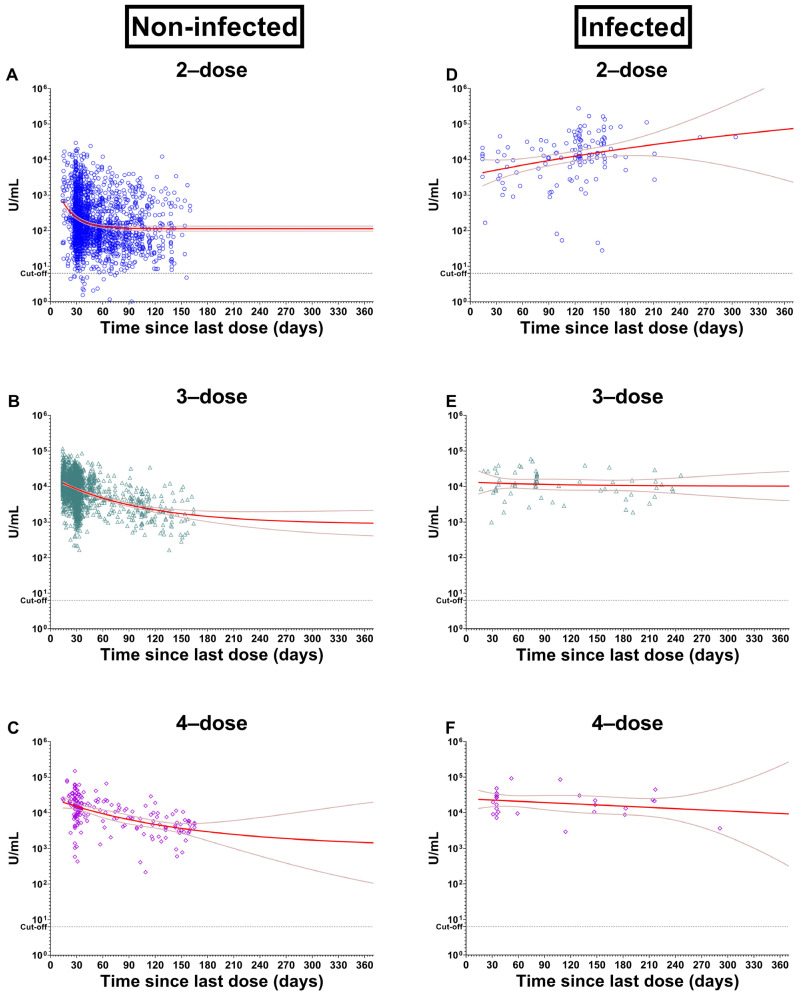
The Ig anti-RBD levels by vaccination dose and history of infection. This figure shows the relation between antibody levels and time since each group’s last dose (days). Non-infected; (**A**) 2-dose, (**B**) 3-dose, and (**C**) 4-dose. Infected; (**D**) 2-dose, (**E**) 3-dose, and (**F**) 4-dose. Trendlines are based on a one-phase exponential decay assumption.

Among the two-dose vaccinated uninfected participants (Figure 2A), the trend line of antibody level showed a rapid decline between 14–45 days since the last dose vaccination and remained steady afterward. The estimated half-life of antibody elicited by 2-dose vaccination was 12.33 days. The antibody also gradually declined over time among the 3-dose and 4-dose vaccinated uninfected individuals. The estimated half-life of antibody was 69.86 and 107.1 days for 3-dose and 4-dose vaccination respectively, which were longer than that acquired after the second dose vaccination (Figure 2B,C).

**Figure 3:**
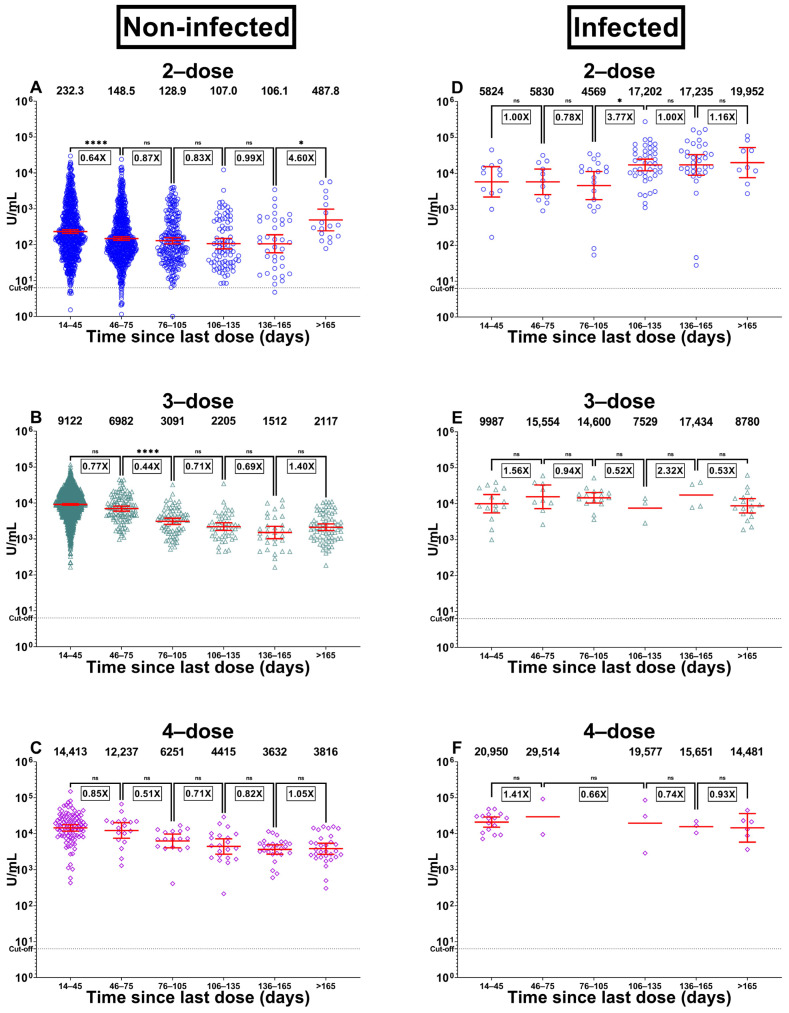
The Ig anti-RBD levels by vaccination dose and history of infection. This figure shows the relation between antibody levels and time since each group’s last dose (days). Non-infected; (**A**) 2-dose, (**B**) 3-dose, and (**C**) 4-dose. Infected; (**D**) 2-dose, (**E**) 3-dose, and (**F**) 4-dose. A log-transformed data was used and converted to a geometric mean ratio (GMR) with a Bonferroni adjustment. The statistically significant symbol denotes; *p* < 0.05 (*) and *p* < 0.0001 (****). *p* > 0.05 is no statistical significance (ns).

From a pooled result classified by the time since last dose vaccination (Table S1 and Figure 3), Ig anti-RBD of two-dose vaccinated uninfected participants was significantly decreased from 14–45 days to 46–75 days post-vaccination [232.3 (206.1, 261.9) versus 148.5 (132.2, 166.7), Geometric mean ratios (GMR) = 0.64, *p* < 0.001]. After 75 days onwards, the antibody remained stable. Similarly, Ig anti-RBD of three-dose vaccinated uninfected participants was significantly decreased from 14–45 days to 46–75 days post-vaccination [9122 (8699, 9566) vs. 6982 (5942, 8204), GMR = 0.77, *p =* 0.037], and 46–75 days to 76–105 days post-vaccination [6982 (5942, 8204) vs. 3091 (2528, 3779), GMR = 0.44, *p* < 0.001].

### 3.3. Immunity in Participants with the Previous Infection

There were 220 participants who had a history of infection by RT-PCR or antigen test. Among the 220 vaccinated infected participants, the median (IQR) Ig anti-RBD level was 11,689 (8787, 15,549) U/mL after the second dose (*n* = 126), 11,541 (9225, 14,438) U/mL after the third dose (*n* = 64) and 19,205 (14,145, 26,075) U/mL (*n* = 30) after the fourth dose as shown in Table 1. There was no significant difference in the antibody levels after two-, three-, and four-dose vaccination in the vaccinated infected participants.

Among the two-dose vaccinated infected participants (Figure 2D), the trend line of antibody increased over time. This is likely because the natural infection occurs after two-dose vaccination, thus boosting the Ig anti-RBD levels to a higher level compared to the vaccinated uninfected counterparts over time (Figure 2A). The trend lines of antibody levels for 3-dose and 4-dose vaccinated infected participants were stable (Figure 2E,F) at significantly higher levels than those achieved by vaccination alone (Figure 2B,C).

From a pooled result and classified by the time since the last dose vaccination (Table S1 and Figure 3). Ig anti-RBD of two-dose vaccinated infected participants remained stable between 14–45 days to 46–75 days and 106–135 days to >165 days. However, between 76–105 days and 106–135 days, the Ig anti-RBD levels were significantly increased [4569 (1856, 11,251) vs. 17,202 (11,846, 24,980), GMR = 3.77, *p* = 0.035]. This is likely due to the natural infection that occurred after two-dose vaccination. There were no significant differences among the 3-dose and 4-dose groups for each period (Table S1). Comparisons of geometric mean ratios of Ig anti-RBD between groups of participants at each timepoint were demonstrated in Table S2.

## 4. Discussion

In this study, we quantified the Ig anti-RBD levels to gain comprehensive insight into SARS-CoV-2 antibody dynamics following vaccination alone or vaccination plus infection (hybrid immunity) in a large population residing in Bangkok, Thailand, across the Alpha (April to July 2021), Delta (August 2021–November 2021), and Omicron variant period (November 2021–December 2022). Natural infection combined with 2–4 vaccines provided a higher and more stable level of Ig anti-RBD over one year than those achieved by vaccination alone. This coincides with our previous study which showed that breakthrough infection after the two-dose CoronaVac vaccination induced a higher level of Ig anti-RBD than two-dose CoronaVac vaccination alone and the third dose vaccination with AZD1222 but comparable to those achieved by the third dose BNT162b2 booster [16,17,18,19]. A large cohort in Monaco also showed that hybrid immunity induces high levels of neutralizing antibody against SARS-CoV-2 [27]. Another study in Denmark which compared between fully BNT162b2 vaccinated individuals with and without previous SARS-CoV-2 infection also found that the proportion of individuals with high levels of total SARS-CoV-2 antibodies was significantly higher in those with prior infection (72.2%) than in infection-naïve individuals (35.4%), indicating robust humoral response induced by hybrid immunity [28]. In addition, a study from a large cohort in Sweden also showed hybrid immunity providing a high degree of protection against COVID-19 reinfection and hospitalisation [8].

Recent evidence has shown that Omicron infections were less severe with a 6.2-fold lower mortality rate compared to Delta infections [10]. Nevertheless, the Omicron variants are more capable of evading the immune response induced by vaccination [6,7]. The two-dose Wuhan-derived vaccine effectiveness is low against the Omicron-variant [7], with rapidly waning humoral immunity induced by two-dose vaccination [25,26]. A third dose booster vaccination was necessary to boost the immune response and increase protection against symptomatic COVID-19 infections [9,13,14,15]. Recent studies that examine the dynamics of neutralizing antibody levels induced by two-dose and three-dose vaccination in infection-naïve individuals also showed that the durability of antibody improves after three-dose vaccination [27]. Besides, individuals reporting prior infection with SARS-CoV-2 exhibited significantly higher neutralizing titres compared to those without breakthrough infection [27]. Even if more than 70–80% of Thai individuals have been infected with COVID-19 and over 80% have received two-dose vaccination (i.e., the majority of the population have hybrid immunity), reinfection still occurs as new variants continue to evolve and evade the host immunity. COVID-19 will most likely become another seasonal respiratory disease like influenza where the vaccine campaigns are promoted based on seasons and probably be most effective when circulating viruses are well-matched with viruses contained in vaccines. Taking the Thai influenza vaccine campaigns starting in June of each year [29] as an example and based on the stable levels of IgG over six months from hybrid immunity in the current study, infected individuals who received at least two-dose vaccination could receive a booster annually before the outbreak season. The annual booster vaccine should be highly recommended among the high-risk group (i.e., pregnant women, elderly individuals (65 years), individuals with chronic medical conditions and healthcare workers.) similar to the recommendation for seasonal influenza immunisation. Locating in the tropical region, Thailand experiences a peak incidence of respiratory disease outbreaks during the rainy season (June to September) [30]. This is because the rainy season creates a conducive environment for the growth and spread of viruses that cause respiratory illnesses [30]. In addition, the first semester of the school year in Thailand usually starts in June, which coincides with the onset of the rainy season. This may increase the risk of transmission of respiratory diseases, as students are in close proximity to each other in classrooms and other school settings. Therefore, COVID-19 vaccination should be given annually by June of each year to prevent outbreaks.

The strength of this study is the large sample size of vaccinated and/or infected individuals over a long period of time. A few limitations included the lack of an exact date of infection and medical records of co-morbidities. The infection was informed by the history of antigen testing or PCR testing without evidence of laboratory confirmation. The number of individuals who received four doses of the vaccine, and infected groups are small. There could be selection bias as participants who sought blood sampling were middle-aged to elderly. Besides, the immune response to vaccinations could be affected by sex, gender, co-morbidities and types of vaccination.

## 5. Conclusions

In conclusion, two-dose vaccinated individuals without natural infection had low levels of Ig anti-RBD, which waned over time. To restore the waning antibody, the third-dose vaccination is recommended for two-dose vaccinated, uninfected individuals. When COVID-19 has transformed into a seasonal respiratory disease like influenza, COVID-19 vaccination should be given annually to prevent outbreaks.

## Figures and Tables

**Table 1:** Demographic characteristics and antibody levels of participants in the study.

	2–Dose	3–Dose	4–Dose	*p*-Value
No history of infection (*n*, 3906)				
*n*	1836	1859	211	
Male (%)	562 (30.6)	482 (25.9)	83 (39.3)	<0.001 *
Age, mean (SD)	41.49 (±14.01)	41.68 (±13.04)	47.73 (±15.79)	**<0.001 ^†^**
Ig anti-RBD, GMT (95%CI) U/mL	173.4 (160.9, 186.9)	7599 (7249, 7967)	8199 (7025, 9568)	**<0.001 ^¶^**
Time since last dose vaccination (days), median (IQR)	41.0 (31.0, 68.0)	29.0 (23.0, 35.0)	60.0 (30.0, 144.0)	**<0.001 ^§^**
14–45 days, median (IQR)	31.0 (29.0, 33.0)	28.0 (21.0, 31.0)	29.5 (28.0, 32.3)	**<0.001 ^§^**
46–75 days, median (IQR)	49.0 (42.0, 59.0)	53.0 (49.0, 62.0)	61.5 (54.8, 66.0)	**<0.001 ^§^**
76–105 days, median (IQR)	90.0 (83.0, 98.0)	95.0 (90.0, 103.3)	89.0 (81.0, 99.0)	**<0.001 ^§^**
106–135 days, median (IQR)	118.0 (112.5, 125.0)	115.5 (110.3, 126.8)	120.0 (115.5, 129.5)	0.226 ^**§**^
136–165 days, median (IQR)	139.0 (138.0, 149.0)	150.0 (139.0, 154.0)	152.5 (145.0, 159.0)	**<0.001 ^§^**
>165 days, median (IQR)	190.0 (176.0, 224.0)	189.0 (182.0, 212.3)	183.0 (173.0, 207.0)	0.341 ^**§**^
Had history of infection (*n*, 220)				
*n*	126	64	30	
Male (%)	125 (32.5)	11 (17.2)	5 (16.7)	0.035 *
Age, mean (SD)	37.33 (±12.75)	39.79 (±12.96)	39.31 (±13.12)	0.821 ^†^
Ig anti-RBD, GMT (95%CI) U/mL	11,689 (8787, 15,549)	11,541 (9225, 14,438)	19,205 (14,145, 26,075)	0.172 ^¶^
Time since last dose vaccination (days), median (IQR)	125.0 (89.75, 145.3)	80.50 (51.50, 171.0)	36.0 (35.0, 148.0)	**0.036 ^§^**
14–45 days, median (IQR)	24.0 (14.0, 33.5)	34.0 (29.0, 37.0)	35.0 (35.0, 35.0)	**0.003 ^§^**
46–75 days, median (IQR)	49.0 (39.0, 57.0)	56.0 (54.5, 67.5)	55.5 (52.0, 59.0)	0.058 ^§^
76–105 days, median (IQR)	91.0 (84.0, 99.0)	80.5 (79.3, 81.0)	N/A	**<0.001 ^§^**
106–135 days, median (IQR)	125.0 (120.0, 126.0)	112.0 (106.0, 130.0)	114.0 (108.0, 130.0)	0.320 ^§^
136–165 days, median (IQR)	151.0 (142.0, 153.0)	156.5 (140.5, 162.8)	148.0 (147.0, 148.0)	0.336 ^§^
>165 days, median (IQR)	202.0 (175.0, 237.0)	216.0 (186.0, 230.0)	215.0 (182.8, 235.5)	0.551 ^§^

* *p*-value from Fisher’s exact test, ^†^ *p*-value from one-way ANOVA, ^§^ *p*-value from Dunn’s test, ^¶^ *p*-value from GMR. The bold value in the *p*-Value column is statistically significant.

## Data Availability

The data presented in this study are available on request from the corresponding author.

## Supplementary Materials

The following supporting information can be downloaded at: https://www.mdpi.com/article/10.3390/vaccines11071184/s1, Table S1: Antibody levels stratified by the interval from the last dose vaccination and blood sampling; Table S2: Geometric mean ratios of Ig anti-RBD between groups of participants stratified by status of infection and number of vaccine doses.
