# Supplementary material for: SARS-CoV-2 Antibody Dynamics after COVID-19 Vaccination and Infection: A Real-World Cross-Sectional Analysis
Source: Vaccines (Basel). 2023 Jun 30;11(7):1184. doi: 10.3390/vaccines11071184 (PMC10384814; doi:10.3390/vaccines11071184)
Supplement: Supplementary file 1 [file vaccines-11-01184-s001.zip › vaccines-2430990-supplementary.pdf]

Table S1. Antibody levels stratified by the interval from the last dose vaccination and blood sampling. The antibody was classified by time since the last dose (days). GMR calculation is performed using the preceding timeline as a referent.

	**2-dose**	**3-dose**	**4-dose**
No history of infection (*n*, 3748)			
Pooled	171.1 (159.3, 185.1)	8013 (7646, 8398)	9353 (7935, 11,026)
14-45 days	232.3 (206.1, 261.9)	9122 (8699, 9566)	14,413 (11,738, 17,696)
	**0.64X, *p*<0.001**	**0.77X, *p*0.037**	0.85X, *p*>0.999
46-75 days	148.5 (132.2, 166.7)	6982 (5942, 8204)	12,237 (7431, 20,152)
	0.87X, *p*>0.999	**0.44X, *p*<0.001**	0.51X, *p*0.432
76-105 days	128.9 (106.1, 156.6)	3091 (2528, 3779)	6251 (4025, 9708)
	0.83X, *p*>0.999	0.71X, *p*0.530	0.71X, *p*>0.999
106-135 days	107.0 (76.56, 149.4)	2205 (1722, 2822)	4415 (2714, 7181)
	0.99X, *p*>0.999	0.69X, *p*0.808	0.82X, *p*>0.999
136-165 days	106.1 (59.44, 189.5)	1512 (1017, 2249)	3632 (2713, 4863)
	**4.60X, *p*0.019**	1.40X, *p*0.783	1.05X, *p*>0.999
> 165 days	487.8 (242.4, 981.7)	2117 (1714, 2249)	3816 (2696, 5400)
Had history of infection (*n*, 220)			
Pooled	11,689 (8787, 15,549)	11,541 (9225, 14,438)	19,205 (14,145, 26,075)
15-45 days	5824 (2202, 15,404)	9987 (5553, 17,963)	20,950 (15,096, 29,074)
	1.00X, *p*>0.999	1.56X, *p*>0.999	1.41X, *p*>0.999
46-75 days	5830 (2574, 13,205)	15,554 (7267, 33,291)	29,514*
	0.78X, *p*>0.999	0.94X, *p*>0.999	
76-105 days	4569 (1856, 11,251)	14,600 (10,410, 20,478)	0.66X, *p*>0.999
	**3.77X, *p*0.035**	0.52X, *p*>0.999	
106-135 days	17,202 (11,846, 24,980)	7529 (931.6, 60,850)	19,577*
	1.00X, *p*>0.999	2.32X, *p*>0.999	0.74X, *p*>0.999
136-165 days	17,235 (8907, 33,350)	17,434 (4369, 69,572)	15,651*
	1.16X, *p*>0.999	0.53X, *p*>0.999	0.93X, *p*>0.999
> 165 days	19,952 (7597, 52,396)	8780 (5540, 13,915)	14,481 (5799, 36,159)

* n less than 4 were not calculated the 95%CI. The bold value is statistically significant.

Table S2. Geometric mean ratios of Ig anti-RBD between groups of participants stratified by status of infection and number of vaccine doses. The antibody was classified by time since last dose (days).

	**2-dose**	**3-dose vs 2-dose, GMR, *p*-value**	**3-dose**	**4-dose vs 3-dose, GMR, *p*-value**	**4-dose**	**4-dose vs 2-dose, GMR, *p*-value**
No history of infection (*n*, 3748)						
Pooled	171.1 (159.3, 185.1)	**46.67X, *p*<0.001**	8013 (7646, 8398)	1.17X, *p*0.428	9353 (7935, 11,026)	**54.45X, *p*<0.001**
15-45 days	232.3 (206.1, 261.9)	**39.26X, *p*<0.001**	9122 (8699, 9566)	**1.58X, *p*0.001**	14,413 (11,738, 17,696)	**62.09X, *p*<0.001**
46-75 days	148.5 (132.2, 166.7)	**47.00X, *p*<0.001**	6982 (5942, 8204)	1.75X, *p*0.414	12,237 (7431, 20,152)	**82.41X, *p*<0.001**
76-105 days	128.9 (106.1, 156.6)	**24.00X, *p*<0.001**	3091 (2528, 3779)	2.02X, *p*0.169	6251 (4025, 9708)	**48.53X, *p*<0.001**
106-135 days	107.0 (76.56, 149.4)	**20.61X, *p*<0.001**	2205 (1722, 2822)	2.00X, *p*0.133	4415 (2714, 7181)	**41.30X, *p*<0.001**
136-165 days	106.1 (59.44, 189.5)	**14.26X, *p*<0.001**	1512 (1017, 2249)	**2.40X, *p*0.038**	3632 (2713, 4863)	**34.20X, *p*<0.001**
> 165 days	487.8 (242.4, 981.7)	**4.34X, *p*<0.001**	2117 (1714, 2249)	**1.80X, *p*0.019**	3816 (2696, 5400)	**7.82X, *p*<0.001**
Had history of infection (*n*, 220)						
Pooled	11,689 (8787, 15,549)	0.99X, *p*>0.999	11,541 (9225, 14,438)	1.66X, *p*0.271	19,205 (14,145, 26,075)	1.64X, *p*0.217
15-45 days	5824 (2202, 15,404)	1.71X, *p*0.622	9987 (5553, 17,963)	2.10X, *p*0.195	20,950 (15,096, 29,074)	**3.60X, *p*0.011**
46-75 days	5830 (2574, 13,205)	2.67X, *p*0.220	15,554 (7267, 33,291)	1.90X, *p*>0.999	29,514*	5.06X, *p*0.249
76-105 days	4569 (1856, 11,251)	**3.20X, *p*0.018**	14,600 (10,410, 20,478)	N/A	N/A	N/A
106-135 days	17,202 (11,846, 24,980)	0.44X, *p*0.781	7529 (931.6, 60,850)	2.60X, *p*>0.999	19,577*	1.14X, *p*>0.999
136-165 days	17,235 (8907, 33,350)	1.01X, *p*>0.999	17,434 (4369, 69,572)	0.90X, *p*>0.999	15,651*	0.91X, *p*>0.999
> 165 days	19,952 (7597, 52,396)	0.44X, *p*0.171	8780 (5540, 13,915)	1.65X, *p*0.909	14,481 (5799, 36,159)	0.73X, *p*>0.999

* n less than 4 were not calculated the 95%CI. The bold is statistically significant.
